
JOURNAL INFORMATION
==============================
NLM Title Abbreviation: J Vet Intern Med
Journal ID: J Vet Intern Med
NLM Journal ID: 8708660
Journal ID: JVIM

Journal of Veterinary Internal Medicine

ISSN: 0891-6640
EISSN: 1939-1676
Publisher: Oxford University Press

ARTICLE INFORMATION
==============================
PMCID: PMC11474866
DOI: 10.1111/jvim.16751
Article ID: JVIM16751
Article version: 1
Subjects: Issue Information, Issue Information

Issue Information

Electronic publication date: 2024 Jan 21
Publication date: 2024 Jan-Feb
Volume: 38
Issue: 1
First page: 1
Last page: 9
Copyright: © 2024 The Authors. Journal of Veterinary Internal Medicine published by Wiley Periodicals LLC on behalf of American College of Veterinary Internal Medicine.
License: This is an open access article under the terms of the http://creativecommons.org/licenses/by/4.0/ License, which permits use, distribution and reproduction in any medium, provided the original work is properly cited.
License URL: https://creativecommons.org/licenses/by/4.0/

Figure count: 0
Table count: 0
Page count: 1

source-schema-version-number: 2.0
cover-date: January/February 2024
details-of-publishers-convertor: Converter:WILEY_ML3GV2_TO_JATSPMC version:6.4.9 mode:remove_FC converted:15.10.2024

==============================

Volume 38, Number 1

January/February 2024

### JOURNAL OF VETERINARY INTERNAL MEDICINE

Open Access

ACVIM

American College of Veterinary Internal Medicine

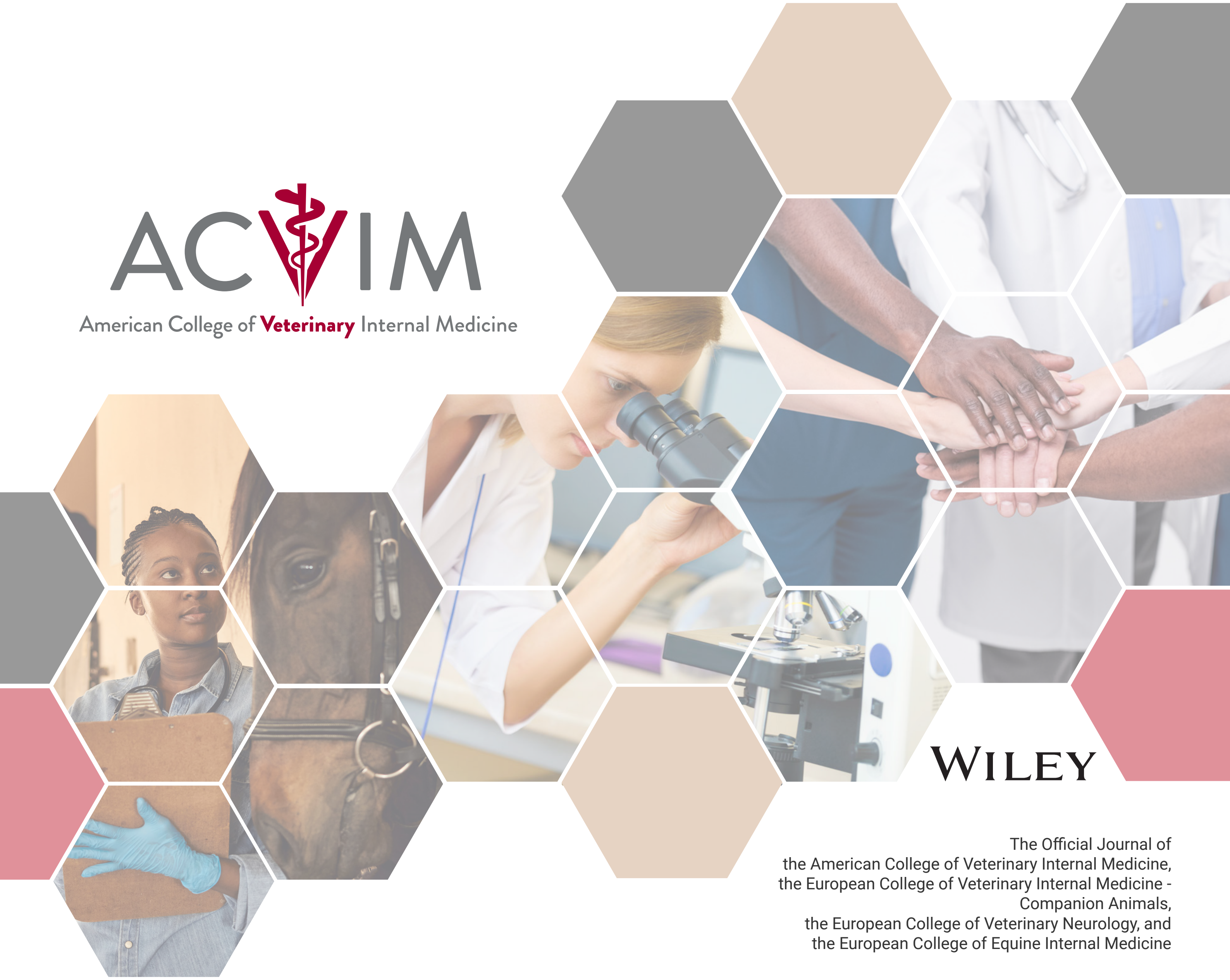

WILEY

The Official Journal of
the American College of Veterinary Internal Medicine,
the European College of Veterinary Internal Medicine - Companion Animals,
the European College of Veterinary Neurology, and
the European College of Equine Internal Medicine

### Journal of Veterinary Internal Medicine

Open Access

ACVIM American College of Veterinary Internal Medicine

The Official Journal of the American College of Veterinary Internal Medicine,
the European College of Veterinary Internal Medicine - Companion Animals, the European College of Veterinary Neurology, and the European College of Equine Internal Medicine

**MISSION STATEMENT**

The mission of the *Journal of Veterinary Internal Medicine* is to advance veterinary medical knowledge and improve the lives of animals by publication of authoritative scientific articles of animal diseases.

**Volume 38, Number 1** **January/February 2024**

LETTERS TO THE EDITOR

10 **Call to action: Accrediting body for graduate veterinary medical education**
*Jane E. Sykes*

12 **Letter regarding "Benign ureteral obstruction in cats: Outcome with medical management"**
*Lillian Aronson, Chad Schmiedt, Joe Bartges, Eric Monnet, and Robert Hardie*

14 **Response to letter regarding "Benign feline ureteral obstruction in cats: Outcome with medical management"**
*Marilyn Dunn, Isabelle Merindol, and Catherine Vachon*

16 **Letter regarding "ACVIM consensus statement on pancreatitis in cats"**
*Penny Watson*

17 **Response to letter regarding "ACVIM consensus statement on pancreatitis in cats"**
*Marnin A. Forman, Joerg M. Steiner, P. Jane Armstrong, Melinda S. Camus, Lorrie Gaschen, Steve L. Hill, Caroline S. Mansfield, and Katja Steiger*

EDITORIAL

18 **Comments from the editors on the term "standard of care"**
*Stephen P. DiBartola and Ken W. Hinchcliff*

CONSENSUS STATEMENT

19 **ACVIM Consensus Statement on the management of status epilepticus and cluster seizures in dogs and cats**
*Marios Charalambous, Karen Muñana, Edward E. Patterson, Simon R. Platt, and Holger A. Volk*

SMALL ANIMAL

Standard Articles

*Cardiology*

41 **Clinical relevance of serum ionized magnesium concentration in dogs with myxomatous mitral valve disease**
*Ock-Kyu Kim, Keon Kim, Sinwook Park, Hayoung Yang, Hyunwoo Kim, Woong-Bin Ro, and Chang-Min Lee*

51 **Efficacy of a mitral regurgitation severity index to predict long-term outcome in dogs with myxomatous mitral valve disease**
*Michelle Vereb, Clarke E. Atkins, Darcy Adin, Thomas Blondel, Melissa Coffman, Seunggon Lee, Emilie Guillot, and Jessica L. Ward*

61 **Aortic annular plane systolic excursion in cats with hypertrophic cardiomyopathy**
*Luke C. Dutton, Ilaria Spalla, Joonbum Seo, Joel Silva, and Jose Novo Matos*

71 **Urine sodium concentration after intravenous furosemide in dogs with acute congestive heart failure and correlation with treatment efficacy**
*Victoria Convey, Terry Huh, Erin J. Achilles, Laura K. Massey, Victoria F. McKaba, Kerry A. Loughran, Marc S. Kraus, Anna R. Gelzer, Alexandra V. Crooks, and Mark A. Oyama*

*Endocrinology*

81 **Relationship between thyroid function and sex hormones in female German shepherd dogs**
*Paola Scarpa, Filippo Tagliasacchi, Francesca Iavazzo, Michela Beccaglia, Adriano Monino, Paolo Dri, and Gianpaolo Milite*

93 **Prevalence of eunatremic, eukalemic hypoadrenocorticism in dogs with signs of chronic gastrointestinal disease and risk of misdiagnosis after previous glucocorticoid administration**
*Antonio Maria Tardo, Francesca Del Baldo, Rodolfo Oliveira Leal, Giorgia Galiazzo, Marco Pietra, Alba Gaspardo, and Federico Fracassi*

102 **Effect of 5′-adenosine monophosphate-activated protein kinase agonists on insulin and glucose dynamics in experimentally induced insulin dysregulation in horses**
*Erin F. Pinnell, Laura D. Hostnik, Mauria R. Watts, Kathryn J. Timko, Allison A. Thriffiley, Mercedes R. Stover, Lauren E. Koenig, Olivia M. Gorman, Ramiro E. Toribio, and Teresa A. Burns*

111 **Thyroid function tests during nonthyroidal illness syndrome and recovery in acutely ill dogs**
*Timothy A. Bolton, David L. Panciera, Caylie D. Voudren, and Matthew I. Crawford-Jennings*

123 **Serum gastrin concentrations in dogs with primary hyperparathyroidism**
*Julieann Vose, Jared Jaffey, Camille Akin, Alexander Spitzer, Barry DeCicco, Enass Bassiouny, Ashley LaClair, Brian Petroff, Jean Brudvig, and Harry Cridge*

130 **The impact of single-dose trazodone administration on plasma endogenous adrenocorticotropic hormone and serum cortisol concentrations in healthy dogs**
*Morgan Brown, Tekla Lee-Fowler, Ellen N. Behrend, and Megan Grobman*

*Genetics*

135 **Precision medicine using whole genome sequencing identifies a novel *dystrophin* (*DMD*) variant for X-linked muscular dystrophy in a cat**
*G. Diane Shelton, Fabrizio Tucciarone, Ling T. Guo, Lyndon M. Coghill, and Leslie A. Lyons*

*Gastroentrology*

145 **Use of octreotide for the treatment of protein-losing enteropathy in dogs: Retrospective study of 18 cases**
*Sara A. Jablonski, Allison S. W. Mazepa, and M. Katherine Tolbert*

152 **Giardiasis and diarrhea in dogs: Does the microbiome matter?**
*Sharon Kuzi, Soha Zgairy, Barbara A. Byrne, Jan Suchodolski, Sondra C. Turjeman, So Young Park, Itamar Aroch, Mike Hong, Omry Koren, and Eran Lavy*

*Hematology*

161 **Retrospective evaluation of hematological ratios in canine parvovirosis: 401 cases**
*Andrea González-Domínguez, José Ignacio Cristobal-Verdejo, Clara López-Espinar, Sergio Fontela-González, Sergio Vázquez, Javier Justo-Domínguez, Jonatan González-Caramazana, Mónica Bragado-Cuesta, Alicia Álvarez-Punzano, and Vicente J. Herrería-Bustillo*

*Hepatology*

167 **Characterization of clinical presentation, histological features, ultrasonographic findings, and survival in 29 dogs with granulomatous hepatitis**
*Kayla D. Prentice, Julie E. Callahan-Clark, Nicola M. Parry, Leslie A. Schwarz, and Cynthia R. L. Webster*

176 **Concurrent hepatopathy in dogs with gallbladder mucocele: Prevalence, predictors, and impact on long-term outcome**
*Sara A. Jablonski, Yue Xiang (Polly) Chen, Jarod E. Williams, Jessica A. Kendziorski, and Rebecca C. Smedley*

*Immunology*
187 **Immunoreactivity of canine, feline, and equine D-dimer with antibodies to human D-dimer**
*Juliet E. Brown, Amir H. Noormohammadi, and Natalie F. Courtman*

*Nephrology/Urology*
197 **Use of molidustat, a hypoxia-inducible factor prolyl hydroxylase inhibitor, in chronic kidney disease-associated anemia in cats**
*Samuel Charles, Ricarda Süssenberger, Terry Settje, Catherine Langston, and Chantal Lainesse*

205 **Renal amyloid-A amyloidosis in cats: Characterization of proteinuria and biomarker discovery, and associations with kidney histology**
*Carlo Palizzotto, Felippo Ferri, Carolina Callegari, Francesco Rossi, Marcello Manfredi, Laura Carcangiu, Gabriele Gerardi, Silvia Ferro, Laura Cavicchioli, Elizabeth Müller, Marco Weiss, Anne-Catherine Vogt, Francesca Lavatelli, Stefano Ricagno, Karyl Hurley, and Eric Zini*

216 **MicroRNA-126 in dogs with immune complex-mediated glomerulonephritis**
*Ariana D. Cherry, Candice P. Chu, Rachel E. Cianciolo, Jessica A. Hokamp, Sarah A. Jacobson, and Mary B. Nabity*

228 **Factors associated with thrombotic disease in dogs with renal proteinuria: A retrospective of 150 cases**
*Luca Fortuna and Harriet M. Syme*

*Neurology*
238 **Prevalence of nonconvulsive seizures and nonconvulsive status epilepticus in dogs and cats with a history of cluster seizures: A retrospective study**
*Carina Tästensen, Sarah Gutmann, Shenja Loderstedt, Thomas Flegel, Helga Demeny, and Petra Baum*

247 **Outcomes of surgically and conservatively managed thoracolumbar and lumbosacral intervertebral disc herniations in cats**
*Jack A. Amey, Theofanis Liatis, Giunio Bruto Cherubini, Steven De Decker, and Max H. Foreman*

258 **Association of the *FGF4L2* retrogene with fibrocartilaginous embolic myelopathy in dogs**
*Colleen Embersics, Danika Bannasch, Kevin Batcher, Elizabeth C. Boudreau, Molly Church, Andrew Miller, Simon Platt, Jey Koehler, Natasha Olby, John Rossmeisl, Daniel Rissi, Robert Grahn, Jonas Donner, and Peter J. Dickinson*

268 **The value of a head turn in neurolocalization**
*Aran Nagendran, Roberto José López, Anna Suñol, Josep Brocal, and Rita Gonçalves*

277 **Polioencephalopathy in Eurasier dogs**
*Faye Rawson, Matthias Christen, Jeremy Rose, Emilie Paran, Tosso Leeb, and Angela Fadda*

*Nutrition*
285 **Long-term safety of dietary salt: A 5-year ProspEctive rAndomized bliNded and controlled stUdy in healThy aged cats (PEANUT study)**
*Brice S. Reynolds, Valerie Chetboul, Jonathan Elliott, Jeremy Laxalde, Patrick Nguyen, Isabelle Testault, Laëticia Dorso, Jérôme Abadie, Hervé P. Lefebvre, and Vincent Biourge*

*Oncology*
300 **Serum thymidine kinase 1 protein concentrations and presence of its autoantibody as biomarkers for screening dogs with malignant tumors**
*Yoonhee Kim, Jeongjoo Park, Byunggak Kim, Kyung-Rok Yu, Hong Sook Kim, Yein Oh, Kyoungwon Seo, Minok Ryu, Chul Park, Dongha Bhang, Ulsoo Choi, and Hwayoung Youn*

308 **Evaluation of the clinical outcome of hypercalcemia of malignancy and concurrent azotemia in dogs with lymphoma**
*Alyssa A. Strumpf, Laura Selmic, and Brian Husbands*

316 **Use of genome-wide DNA methylation analysis to identify prognostic CpG site markers associated with longer survival time in dogs with multicentric high-grade B-cell lymphoma**
*Yong Bin Teoh, Teita Ishizaki, Yumiko Kagawa, Shoko Yokoyama, Jaroslav Jelinek, Yuki Matsumoto, Hirotaka Tomiyasu, Hajime Tsujimoto, Mitsuyoshi Takiguchi, and Jumpei Yamazaki*

*Pharmacology*

326 **Tolerability of long-term cannabidiol supplementation to healthy adult dogs**
*Isabella Corsato Alvarenga, Kim M. Wilson, and Stephanie McGrath*

*Respiratory*

336 **Lung ultrasound score in dogs and cats: A reliability study**
*Stefano Oricco, Daniele Medico, Ilaria Tommasi, Richard Marcello Bini, and Roberto Rabozzi*

Case Reports

*Endocrinology*

346 **Acute adrenal necrosis in a young female cat**
*Rebecca A. Manson, Tara N. Hammond, and Julie E. Callahan Clark*

351 **Transsphenoidal hypophysectomy for the treatment of hypersomatotropism secondary to a pituitary somatotroph adenoma in a dog**
*Matthew M. E. Steele, Jack S. Lawson, Christopher Scudder, Alice H. Watson, Nicola T. Z. Ho, Dylan Yaffy, Daniel Batchelor, and Joe Fenn*

*Hematology*

358 **Discordance between *ABC* blood phenotype and genotype in a domestic short-haired cat**
*Argyrios Ginoudis, Bertrand Canard, Alexandra Kehl, Urs Giger, Christos Koutinas, Stella Christoforaki, Erasmia Smyroglou, Zoe Polizopoulou, and Mathios E. Mylonakis*

*Infectious Disease*

363 **Clinical and genomic features of *Listeria monocytogenes*-associated mesenteric lymphadenitis in a cat**
*Miguel Garcia-de la Virgen, Inmaculada López-Almela, Alexandra Moura, Sergio Vázquez, Sara Perez-Montagud, Alexandre Leclercq, Marc Lecuit, and Juan J Quereda*

*Nephrology/Urology*

370 **Uroliths composed of antiviral compound GS-441524 in 2 cats undergoing treatment for feline infectious peritonitis**
*Marissa Allinder, Beth Tynan, Cara Martin, Amelia Furbish, Glenn Austin, Joe Bartges, and Bianca N. Lourenço*

Perspective

*General*

375 **Lessons learned: Shaping the evolution of veterinary specialty education**
*Jane E. Sykes*

Pilot Study

*Pharmacology*

381 **Pharmacodynamic effects of molidustat on erythropoiesis in healthy cats**
*Annette Boegel, Ingo Flamme, Ralph Krebber, Terry Settje, Franziska Schmidt, Eva Kruedewagen, Sandra Mangold-Gehring, Chantal Lainesse, Andreas Moritz, and Gerald Beddies*

**EQUINE**

Standard Articles

*Ophthalmology*

388 **Pain scoring systems in hospitalized horses with ocular disease**
*Dayna Jodzio, Sally DeNotta, Caryn Plummer, and Chris Sanchez*

*Cardiology*

398 **Effect of physiological and pharmacological stress on heart rate, blood pressure, and echocardiographic measurements in healthy Warmblood horses**
*Alexander Dufourni, Eva Buschmann, Ingrid Vernemmen, Glenn Van Steenkiste, Gunther van Loon, and Annelies Decloedt*

411 **Intravenous loss of over-the-wire catheter guidewires in 13 horses**
*Kallie J. Hobbs, Kimberly A. S. Young, Sara Nannarone, Daniela Luethy, Charlotte Hopster-Iversen, Harold C. McKenzie, and Elsa K. Ludwig*

*Genetics*

417 **Genetic polymorphisms in vitamin E transport genes as determinants for risk of equine neuroaxonal dystrophy**
*Yunzhuo Ma, Sichong Peng, Callum G. Donnelly, Sharmila Ghosh, Andrew D. Miller, Kevin Woolard, and Carrie J. Finno*

*Immunology*

424 **Effect of dexamethasone on antibody response of horses to vaccination with a combined equine influenza virus and equine herpesvirus-1 vaccine**
*Nicole Kreutzfeldt, Thomas M. Chambers, Stephanie Reedy, Kennedy Michelle Spann, and Nicola Pusterla*

*Neurology*

431 **Clinical and histopathological features in horses with neuroaxonal degeneration: 100 cases (2017-2021)**
*Kara A. Brown, Susan J. Bender, and Amy L. Johnson*

440 **Common and atypical presentations of *Anaplasma phagocytophilum* infection in equids with emphasis on neurologic and muscle disease**
*Monica Aleman, Ujwala Vedavally, Nicola Pusterla, Fiona Wensley, Emily Berryhill, and John E. Madigan*

449 **Juvenile idiopathic epilepsy in Egyptian Arabian foals, a potential animal model of self-limited epilepsy in children**
*Monica Aleman, Ruba Benini, Sami Elestwani, and Tatiana Vinardell*

*Nutrition*

460 **Effects of intravenous administration of ascorbic acid (vitamin C) on oxidative status in healthy adult horses**
*Sandra D. Taylor, Kelsey A. Hart, Sarah Vaughn, Shyla C. Giancola, Priscila B. S. Serpa, and Andrea P. Santos*

*Respiratory*

469 **Effects of soaked hay on lung function and airway inflammation in horses with severe asthma**
*Roxane Westerfeld, Flavie Payette, Valérie Dubuc, Estelle Manguin, Khristine Picotte, Guy Beauchamp, Christian Bédard, and Mathilde Leclere*

477 **Microarray molecular mapping of horses with severe asthma**
*Samuel J. White, Laurent Couetil, Eric A. Richard, Eliane Marti, and Philippe B. Wilson*

485 **Severe asthma in horses is associated with increased airway innervation**
*Laurence Leduc, Mathilde Leclère, Laurie Girardot Gauthier, Olivier Marcil, and Jean-Pierre Lavoie*

495 **Breath characteristics and adventitious lung sounds in healthy and asthmatic horses**
*Eloïse Greim, Jan Naef, Sophie Mainguy-Seers, Jean-Pierre Lavoie, Sophie Sage, Gaudenz Dolf, and Vinzenz Gerber*

**FOOD ANIMAL**

Standard Articles

*Immunology*

505 **The effect of pegbovigrastim administration on the nonspecific immunity of calves**
*Katarzyna Dudek, Ewelina Szacawa, and Dariusz Bednarek*

*Infectious Disease*

**514 Clinical presentation, medical management, and outcomes in 35 hospitalized sheep diagnosed with bluetongue virus disease**
*Lisa Gamsjäger and Munashe Chigerwe*

*Nutrition*

**520 Predictors of blood ionized calcium concentration in sick adult cattle**
*Tolga Karapinar, Kenan Cagri Tumer, Peter D. Constable, and Sébastien M. C. Buczinski*

**ABSTRACTS**

**530 2023 ACVIM Forum Research Abstract Program**

**730 Research Communications of the 33rd ECVIM-CA Congress**

**ERRATUM**

**864 Erratum**

### Journal of Veterinary Internal Medicine

Open Access

ACVIM American College of Veterinary Internal Medicine

The Official Journal of the American College of Veterinary Internal Medicine,
the European College of Veterinary Internal Medicine - Companion Animals, the European College of Veterinary Neurology,
and the European College of Equine Internal Medicine

### EDITORIAL BOARD

Kenneth W. Hinchcliff, BVSc, PhD, DACVIM
Editor-in-Chief
The University of Melbourne
Stephen P. DiBartola, DVM, DACVIM
Editor-in-Chief
The Ohio State University
Joshua Stern, DVM, PhD, DACVIM
Associate Editor - Cardiology
North Carolina State University
Jose Novos Matos, DVM, PhD, DECVIM-CA, AFHEA, MRCVS
Associate Editor - Small Animal Internal Medicine, Cardiology
University of Cambridge
Mark Stevenson, MVSc, PhD, MANZCVSc
Consulting Editor for Experimental Design & Statistics
The University of Mebourne
George Moore, DVM, MS, PhD, DACVIM
Consulting Editor for Experimental Design & Statistics
Purdue University
Kristopher J. Hughes, BVSc (Hons 1), FANZCVS, DECEIM,
Associate ACVIM
ECEIM Associate Editor - Large Animal Internal
Medicine (Equine)
Charles Sturt University
Linda Kidd, DVM, PhD, DACVIM
Associate Editor - Small Animal Internal Medicine
(Infectious Disease)
Linda Kidd Veterinary Internal Medicine Consulting
M. Julia B. Felippe, MV, MSc, PhD, DACVIM
Associate Editor - Large Animal Internal Medicine (Equine)
Cornell University
Shelly L. Vaden, DVM, PhD, DACVIM
Associate Editor - Small Animal Internal Medicine
(Nephrology/Urology)
North Carolina State University
Michael Willard, DVM, DACVIM
Associate Editor - Small Animal Internal Medicine
Texas A&M University
Andrea Tipold, DVM, DECVN
ECVN Associate Editor - Neurology
School of Veterinary Medicine Hannover
Robert Rebhun, DVM, PhD, DACVIM
Associate Editor - Hematology/Oncology
University of California, Davis
Eric Zini, PD, PhD, DECVIM-CA
ECVIM-CA Associate Editor - Small Animal Internal Medicine
(Endocrinology)
University of Zurich & Istituto Veterinario di Novara &
University of Padua
Munashe Chigerwe, BVSc, MPH, PhD, DACVIM
Associate Editor - Large Animal Internal Medicine (Food & Fiber)
University of California, Davis
Lauren Trepanier, DVM, PhD, DACVCP, DACVIM
Associate Editor – Small Animal Internal Medicine (Pharmacology,
Pharmacokinetics, Toxicology, and Therapeutic Trials)
University of Wisconsin
Valerie Parker, DVM, DACVIM
Associate Editor - Small Animal Internal Medicine, Nutrition
The Ohio State University
Steven Friedenberg, DVM, PhD, DACVECC
Associate Editor - Small Animal Internal Medicine
(Genetics, Genomics)
University of Minnesota
Anthony Mutsaers, DVM, PhD, DACVIM
Associate Editor - Oncology
University of Guelph
John Farrelly, DVM, DACVIM, DACVR
Associate Editor - Oncology
Blue Pearl Veterinary Partners

### ACVIM Board of Regents Officers

Joerg Steiner, DMV, PhD, DACVIM, DECVIM-CA
Board Chair
Sarah Gillings, DVM, MS, DACVIM
President
Bill Tyrrell, DVM, DACVIM
Treasurer

### Ex-Officio Member of the ACVIM Board of Regents

Jane Sykes, BVSc (Hons), PhD, MBA, DACVIM
Immediate Past Board Chair

### The Editorial and ACVIM Offices are located at:

116 Inveress Drive East, Suite 375, Englewood,
CO 80112

**Telephone:** 303-231-9933 or 800-245-9081

**Editorial Email:** JVIM@ACVIM.org.
**ACVIM Email:** ACVIM@ACVIM.org.

The Journal is available online at www.wileyonlinelibrary.com/journal/jvim.

### ACVIM Board of Regents Members

Brent Credille, DVM, PhD, DACVIM
Member At-Large
Tracy Hill, DVM, DACVIM, PhD, DECVIM-CA
Member At-Large
Roger A. Hostutler, DVM, MS, DACVIM
President, Small Animal Internal Medicine
Chris Sanchez, DVM, PhD, DACVIM
President, Large Animal Internal Medicine
Steven L. Rosenthal, DVM, DACVIM
President, Cardiology
Joan R. Coates, DVM, MS, DACVIM
President, Neurology
Chad Johannes, DVM, DACVIM
President, Oncology
Shannon Carter, EdD, CAE
Chief Executive Officer

### Journal of Veterinary Internal Medicine

Open Access

ACVIM American College of Veterinary Internal Medicine

The Official Journal of the American College of Veterinary Internal Medicine, the European College of Veterinary Internal Medicine - Companion Animals, the European College of Veterinary Neurology, and the European College of Equine Internal Medicine

Wiley Open Access articles posted to repositories or websites are without warranty from Wiley of any kind, either express or implied, including, but not limited to, warranties of merchantability, fitness for a particular purpose, or non-infringement. To the fullest extent permitted by law Wiley disclaims all liability for any loss or damage arising out of, or in connection with, the use of or inability to use the content.

**Open Access and Copyright**

All articles published by *Journal of Veterinary Internal Medicine* are fully open access: immediately freely available to read, download and share. All articles are published under the terms of a Creative Commons license. Copyright on any research article published in the *Journal of Veterinary Internal Medicine* is retained by the author(s).

Further information about open access licenses and copyright can be found at: https://authorservices.wiley.com/author-resources/Journal-Authors/licensing/licensing-info-faqs.html#4. Information on the licenses used by *Journal of Veterinary Internal Medicine* can be found at: www.wileyonlinelibrary.com/journal/jvim

**Disclaimer**

The Publisher, the American College of Veterinary Internal Medicine and Editors cannot be held responsible for any errors in or any consequences arising from the use of information contained in this journal. The views and opinions expressed do not necessarily reflect those of the Publisher, the American College of Veterinary Internal Medicine or Editors, neither does the publication of advertisements constitute any endorsement by the Publisher, the American College of Veterinary Internal Medicine, Editors, or Authors of the products advertised.

For submission instructions and all other information visit: www.wileyonlinelibrary.com/journal/jvim
